# Supplementary figures and images for: COVID-19 mortality is associated with pre-existing impaired innate immunity in health conditions
Source: PeerJ. 2022 May 6;10:e13227. doi: 10.7717/peerj.13227 (PMC9083528; doi:10.7717/peerj.13227)

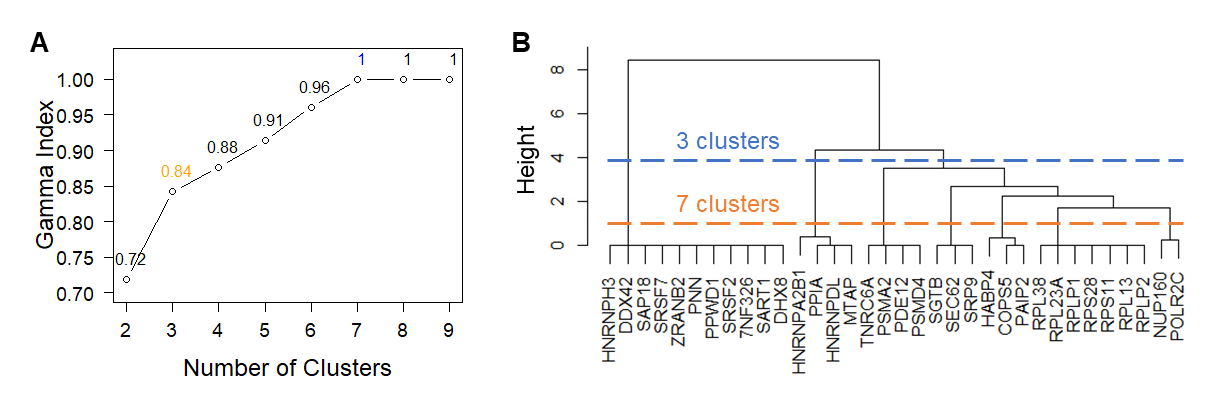

Supplement: Supplemental Information 3 — (A) Goodman-Kruskal-gamma index for different numbers of clusters. The indices corresponding to the 3-cluster and 7-cluster results are colored in orange and blue. (B) Dendrogram of the hierarchical clustering results. [file peerj-10-13227-s003.png]
